# Bonafide, type-specific human papillomavirus persistence among HIV-positive pregnant women: predictive value for cytological abnormalities, a longitudinal cohort study

**DOI:** 10.1590/0074-02760150393

**Published:** 2016-02

**Authors:** Angela RI Meyrelles, Juliana D Siqueira, Pâmela P dos Santos, Cristina B Hofer, Ronir R Luiz, Héctor N Seuánez, Gutemberg Almeida, Marcelo A Soares, Esmeralda A Soares, Elizabeth S Machado

**Affiliations:** 1 Universidade Federal do Rio de Janeiro, Instituto de Ginecologia, Rio de Janeiro, RJ, Brasil; 2 Universidade Federal do Rio de Janeiro, Programa de Pós-Graduação em Ciências Cirúrgicas, Rio de Janeiro, RJ, Brasil; 3 Instituto Nacional de Câncer, Programa de Genética, Rio de Janeiro, RJ, Brasil; 4 Universidade Federal do Rio de Janeiro, Faculdade de Medicina, Rio de Janeiro, RJ, Brasil; 5 Universidade Federal do Rio de Janeiro, Instituto de Puericultura e Pediatria Martagão Gesteira, Rio de Janeiro, RJ, Brasil; 6 Universidade Federal do Rio de Janeiro, Instituto de Estudos em Saúde Coletiva, Rio de Janeiro, RJ, Brasil; 7 Universidade Federal do Rio de Janeiro, Departamento de Genética, Rio de Janeiro, RJ, Brasil

**Keywords:** pregnancy, persistence, HPV, HIV

## Abstract

This study investigated the rate of human papillomavirus (HPV) persistence, associated risk factors, and predictors of cytological alteration outcomes in a cohort of human immunodeficiency virus-infected pregnant women over an 18-month period. HPV was typed through *L1* gene sequencing in cervical smears collected during gestation and at 12 months after delivery. Outcomes were defined as nonpersistence (clearance of the HPV in the 2nd sample), re-infection (detection of different types of HPV in the 2 samples), and type-specific HPV persistence (the same HPV type found in both samples). An unfavourable cytological outcome was considered when the second exam showed progression to squamous intraepithelial lesion or high squamous intraepithelial lesion. Ninety patients were studied. HPV DNA persistence occurred in 50% of the cases composed of type-specific persistence (30%) or re-infection (20%). A low CD4^+^T-cell count at entry was a risk factor for type-specific, re-infection, or HPV DNA persistence. The odds ratio (OR) was almost three times higher in the type-specific group when compared with the re-infection group (OR = 2.8; 95% confidence interval: 0.43-22.79). Our findings show that bonafide (type-specific) HPV persistence is a stronger predictor for the development of cytological abnormalities, highlighting the need for HPV typing as opposed to HPV DNA testing in the clinical setting.

A high prevalence of certain human papillomavirus (HPV) types has been found in cervical cancer (CC) (zur Hausen 2009). The majority of HPV infections is transient and is cleared by the human host (Schmeink et al. 2013). In a minority of cases infection persists, increasing the chance of CC development (Ramanakumar et al. 2010), and several factors are associated with HPV persistence such as use of tobacco, oral contraceptives, and genetic susceptibility to infection (Wang & Hildesheim 2003, Moscicki et al. 2012). HPV replication is essentially intraepithelial with little or no viraemia, therefore there is no inflammation and interferon response is blocked (Stanley 2010). The oncogenic potential of HPV types and variants is also a factor for persistence (Muñoz et al. 2003).

Immunosuppression conditions caused by human immunodeficiency virus (HIV) infection (Kang & Cu-Uvin 2012, Luz et al. 2012) and pregnancy (Palle et al. 2000, Nobbenhuis et al. 2002) are important factors that increase HPV acquisition, persistence, and development of CC. Pregnant women tend to have higher rates of progression from lesion to carcinoma during gestation and after delivery (Palle et al. 2000). Clearance of HPV is lower during the first two trimesters of pregnancy (Nobbenhuis et al. 2002). Interestingly, the additive effect of these two factors has been subject of controversy (Minkoff et al. 2006). There are some laboratory findings suggesting that degradation of p53 by HPV is more efficient when the codon 72 of *TP53* encodes an arginine (Arg) residue. The Arg/Arg genotype was found to be more prevalent in CC biopsies and in individuals with progression from squamous intraepithelial lesions (SIL) to CC (Storey et al. 1998, Habbous et al. 2012), but the role of *TP53* gene polymorphisms on the persistence of HPV infection is still controversy (Klug et al. 2009, Koshiol et al. 2009).

The cellular immunity to HPV involves CD4^+^ T-cell responses and the contribution of CD8^+^ cytotoxic T-cells. The use of combination antiretrovirals is expected to restore CD4^+^ T-cell immunity in HIV-infected individuals, but fails to restore protective HPV-specific immunity because HPV antigen exposure occurs with little inflammation (Van der Burg & Palefsky 2009). On the other hand, Blitz et al. (2013) showed that highly active antiretroviral therapy has a positive impact on clearance of high-risk (HR) HPV types.

There is a large variation in the definition of HPV persistence, both in time interval and in the molecular tests used to identify the infecting HPV. There is also a positive correlation between time span of HPV persistence and the risk of developing CC (Koshiol et al. 2008). Only a few studies addressed true, type-specific HPV persistence over time, especially in low and middle resource settings (Fukuchi et al. 2009, Datta et al. 2012).

We have previously showed the prevalence of HPV infection among HIV-infected pregnant women (Meyrelles et al. 2013). Data on persistence and type of persistence (type-specific vs. re-infection) after pregnancy are scarce in the literature (Nobbenhuis et al. 2002,Jalil et al. 2013). In the present study, we have analysed socio-demographic, clinical, and host/viral genetic factors associated with HPV persistence in this cohort. Clinical and molecular (type-specific) HPV persistence were compared and evaluated as predictors of cytological alteration outcomes over an 18-month period.

## SUBJECTS, MATERIALS AND METHODS

*Subjects and ethics* - This was a prospective, longitudinal cohort study of HPV persistence involving 90 HPV and HIV-infected pregnant women enrolled by the Program for HIV-infected Pregnant Women at Federal University of Rio Janeiro (UFRJ), Rio de Janeiro, Brazil, from April 2009 (1st enrollment) to July 2012 (last 18-month collection). HIV-positive serological status was confirmed by rapid HIV test or ELISA tests and a subsequent Western blot in distinct samples following recommendation by the Brazilian Ministry of Health. All women who agreed to participate signed an informed consent form before enrollment. The study was approved by the ethical committees of both centres involved in the study: UFRJ (protocols: 029/08 - Cle- mentino Fraga Filho University Hospital; 18/10: Mar- tagão Gesteira Childcare and Pediatrics Institute) and the National Cancer Institute (INCA) (protocol: 142/10).

This study was conducted in accordance with the ethical standards of the Brazilian National Ethical Committee as well as the Ethical Committee from the UFRJ and the INCA and with the Helsinki Declaration of 1975, as revised in 1983.

*Samples and study variables* - Cervical smears were collected at two different times from all 90 patients at the second trimester of gestation and 12 months after delivery [average of time interval: 17 (± 4.5) months]. Collection was performed with two endocervical cytobrushes; one was placed in 100% ethanol for Pap smear diagnosis, while the other was placed into 1 ml phosphate-buffered saline and sent to INCA for molecular analyses. A structured questionnaire containing demographic data, gynaecologic and obstetric history, and other risk factors for HPV infection was performed. Additional variables related to HIV infection have also been collected.

*Sample processing and molecular analyses* - Genomic and viral DNA was extracted with the QIAamp DNA mini kit (QIAGEN, USA) following manufacturer’s specifications. A genome fragment of the HPV *L1* gene was polymerase chain reaction (PCR)-amplified in a nested reaction with standard primers and conditions as previously described (Meyrelles et al. 2013). Host *TP53* gene exon 4 (containing codon 72 of the predicted protein sequence) was also PCR-amplified as described elsewhere and used to assess DNA quality and integrity, thus validating the detection of HPV DNA (Meyrelles et al. 2013).

PCR products were purified and sequenced in a 3130XL Genetic Analyzer (Life Technologies, USA). Sequences were assembled and edited with SeqMan (DNAStar, USA). Sequences suggestive of multiple infections (electropherograms containing multiple peaks at definite positions) were subject to cloning into pMOS (GE Healthcare, USA) and transformation into *Escherichia coli* DH5α competent cells according to the manufacturer’s recommendations. An average of 10 bacterial colonies were sequenced and processed as described above to define each of the multiple HPV types.

HPV typing was performed by submitting edited bulk or clonal sequences to the Blast algorithm (ncbi.nlm.nih.gov/blast) and HPV types were assigned to the best hits of the database for each query sequence. Typing was further confirmed by phylogenetic analysis through clustering of query sequences to HPV type references.*TP53* alleles and genotypes were classified by visual inspection of codon 72 of each sequence for the presence of a cytosine (coding for a proline residue) or a guanine (coding for Arg).

*Definition of HPV oncogenic potential and HPV persistence* - The HPV types 16, 18, 26, 30, 31, 33, 34, 35, 39, 45, 51, 52, 53, 56, 58, 59, 66, 67, 68, 69, 73, and 82 were considered as HR types while HPV 6, 11, 40, 42, 43, 44, 54, 61, 70, 72, 81, 89, 32, 62, 74, 83, 84, 86, 87, and 91 were defined as low-risk (LR) types (Muñoz et al. 2003, Várnai et al. 2007).

Nonpersistence was defined as the clearance of the HPV DNA in the second sample collected. Re-infection was defined as the detection of different types of HPV DNA in the two collected samples. Type-specific HPV persistence required the match of the HPV type found in both samples, either as major or minor strains (for the multiply-infected cases). Re-infection cases and type-specific HPV persistence were combined in the group denominated HPV DNA persistence. In case of multiple infections, each type was counted separately for determination of persistence rates.

*Pap smear cytological classification and outcomes* - Cytological results of Pap smears were classified according to the 2001 Bethesda reporting guidelines (Solomon et al. 2002). Outcomes were considered as unfavourable when the second result showed the progression to low SIL (LSIL) or high SIL (HSIL).

*Statistical analyses* - All statistical evaluations were carried out with SPSS v.13. A descriptive analysis for calculating averages, medians, and standard deviations was conducted for continuous variables, while proportions were calculated for categorical variables. Univariate analyses were carried out using independent tests for variables following a normal distribution, and Wilcoxon (Mann-Whitney *U*) two-sample tests for those not following a normal distribution. Categorical variables were analysed with chi-square or Fisher’s exact test. Variables with a p-value ≤ 0.20 in the univariate analyses were included in a logistic regression analysis.

## RESULTS

*General cohort characteristics* - One hundred and forty patients were enrolled. Twenty-eight patients (20%) had no detected HPV DNA in their first sample and were not included in the analysis. In five patients (3.6%), no DNA was recovered in the second sample suggesting lack of cells in the sample, and 17 (12.1%) were lost to follow-up. Ninety patients were analysed.

Clearance of HPV infection occurred in 50% (45 patients), type-specific persistence in 30% (27 patients), and re-infection in 20% (18 patients). There was no difference in the time interval between the first and second sample collection among the three groups.

At study entry, the mean age of the cohort was 27 ± 6 years, and 62.2% were above 25 years of age. Sixty-eight percent were married or had a co-habitating status and 59% had completed primary education. The mean age of the first sexual intercourse was 15 ± 2.4 years, 53.3% being primiparous, and 62.2% had more than three sexual partners during life. Thirty-nine percent were smokers and 52% had used oral contraceptives. Past use of condom was observed in 28%. Twenty-seven percent had reported previous sexually transmitted diseases and 39% had abnormal cervical cytology at entry (atypical squamous cells of undetermined significance, LSIL, or HSIL); this number rose to 44.4% one year after delivery. Regarding HIV-1-related variables, 30% of the patients were on preconceptional combination antiretroviral therapy,the median CD4^+^ T-cell counts at entry were 441 cells/mm^3^ and almost 57% had counts above 350 cells/mm^3^, the median HIV viral load (VL) was 1,085 copies per mL of plasma (Table I).

**TABLE I:** Sociodemographic, clinical, and laboratorial characteristics of 90 human papillomavirus/human immunodeficiency virus (HIV) co-infected pregnant women

Characteristic	n (%)
Age (years) (mean ± SD)	27 ± 6.3
Age ≥ 25 years	56 (62.2)
Married/co-habitating status	61 (67.8)
Complete primary education	53 (58.9)
First sexual intercourse (years) (mean ± SD)	15 ± 2.4
Parity > 1	42 (46.7)
Past/present smoking	35 (38.9)
Number of sexual partners ≥ 4	56 (62.2)
Previous sexually transmitted diseases	24 (26.7)
Past use of oral contraceptive	47 (52.2)
Past use of condom	25 (27.8)
Presence of ASC-US, LSIL, or HSIL in cytological smears at entry	35 (38.9)
Presence of ASC-US, LSIL, or HSIL in cytological smears 1 year after delivery	40 (44.4)
Pre-conception initiation of cART	27 (30)
Median CD4^+^ T-cell counts (cells/mm^3^) at entry (IQR_50_)^*a*^	441 (273-591)
Median CD4^+^ T-cell counts (cells/mm^3^) at delivery (IQR_50_)^*b*^	526 (354-691)
CD4^+^ T-cell counts (cells/mm^3^) > 350 mm^3^/mL^*a*^ at entry	47 (56.6)
CD4^+^ T-cell counts (cells/mm^3^) > 350 mm^3^/mL^*b*^ at delivery	58 (76.3)
Median HIV VL (copies/mL; IQR_50_)^*c*^ at entry	1,085 (78-8,827)
*TP53* codon 72 genotype	
Arg/Arg	35 (38.9)
Arg/Pro	37 (41.1)
Pro/Pro	18 (20)

*a*: missing seven patients; *b*: missing 14 patients; *c*: missing seven patients; Arg: arginine; ASC-US: atypical squamous cells of undetermined significance; cART: combination antiretroviral therapy; HSIL: high squamous intraepithelial lesions; IQR: interquartile range; LSIL: low squamous intraepithelial lesions; SD: standard deviation; VL: viral load.

*TP53* genotypes at codon 72 were distributed as 38.9% Arg/Arg homozygous, 20% Pro/Pro homozygous, and 41.1% Arg/Pro heterozygous.

*HPV typing and persistence* - Twenty-nine different HPV types were identified at baseline (Table II). Of those, 16 were classified as HR and 13 as LR types. Overall, 73 patients (81.1%) were infected by HR types, either as mono or multiple infections. Eighteen patients (20%) had detectable multiple infections and in 15 of those (83.3%) HR types were present.

**TABLE II:** Distribution of human papillomavirus (HPV) types and number of infections in the first sample and their associated persistence at 18 months

HPV type at first sample	Cases (n)t	Type-specific persistence n (%)
**HPV16**	35	9 (25.7)
**HPV58**	11	4 (36.4)
**HPV35**	5	1 (20)
**HPV53**	5	2 (40)
**HPV66**	4	0 (0)
**HPV73**	4	1 (25)
**HPV31**	3	0 (0)
**HPV45**	3	0 (0)
**HPV69**	3	0 (0)
**HPV82**	3	0 (0)
**HPV18**	2	2 (100)
**HPV33**	2	0 (0)
**HPV52**	2	0 (0)
**HPV56**	2	2 (100)
**HPV67**	1	1 (100)
**HPV68**	1	0 (0)
HPV81	6	1 (33.3)
HPV83	6	2 (16.7)
HPV62	5	2 (40)
HPV06	3	1 (33.3)
HPV55	2	0 (0)
HPV61	2	0 (0)
HPV11	1	0 (0)
HPV32	1	0 (0)
HPV42	1	0 (0)
HPV44	1	0 (0)
HPV70	1	1 (100)
HPV72	1	0 (0)
HPV84	1	0 (0)

in boldface: high-risk HPV types.

The most prevalent HR HPV type found at baseline was HPV 16, present in 35 infections (29.9%), followed by HPV 58 (11 cases), HPV 35 and 53 (5 cases), and HPV 73 and 66 (4 cases). HPV 18, consensually recognised as associated to cervical oncogenesis (Muñoz et al. 2003), was only present in two infections (1.7%). With respect to LR types, HPV 83 and HPV 81 were the most prevalent (6 cases each; 5.1%), followed by HPV 62 (5 cases; 4.3%) and HPV 6 (3 cases; 2.6%).

HPV DNA persistence was found in 45 patients (50%): 27 women (30%) presented a type-specific persistence, while re-infection with another HPV type occurred in 18 women (20%). Table II depicts all cases of type-specific persistence. The total number of infections was low for most individual HPV types found and though we were unable to draw conclusions about type-specific persistence efficiency, some interesting results are noteworthy. HPV 16 and 58, the two most common HR types found, persisted in 26 and 36% of the cases, respectively. Several highly oncogenic HR types (e.g., HPV 66, 45, 31, 69 and 82) did not persist in any infection, despite being present in only three to four cases each. Finally, HPV 18 and HPV 56 persisted in the two infections where they were detected at baseline. Regarding the LR types, those with the highest persistence rates were HPV 70 (100%), followed by HPV 62 (40%), HPV 6, and HPV 81 (33.3% each).

*Risk factors for HPV persistence* - We have evaluated factors associated with HPV persistence for the three defined groups (HPV clearance, re-infection, and type-specific persistence (Table III) and odds ratio (OR) for type-specific persistence or re-infection (Table IV). The total number of type-specific persistence or re-infection cases was low, therefore precluding us from performing multivariate analyses. Age of first sexual intercourse > 15 years, past use of oral contraceptive, lack of condom use, CD4^+^ T-cell counts < 350 cells/µL, and a high HIV VL at study entry were associated with HPV DNA persistence. Only CD4^+^ T-cell counts < 350 cells/µL was an independent risk factor for HPV DNA persistence [OR = 4.6; 95% confidence interval (CI): 1.00-21.3].

**TABLE III:** Factors associated with human papillomavirus (HPV) clearance, re-infection, or type-specific persistence (n = 90)

Variable	Clearance (n = 45)	Re-infection (n = 18)	Type-specific persistence (n = 27)	p
		
	n (%)	n (%)	n (%)	
Age ≥ 25 years	26 (57.8)	10 (55.6)	20 (74.1)	0.312
Age of first sexual intercourse ≤ 15 years	33 (73.3)	10 (55.6)	13 (48.1)	0.083
Parity > 1	23 (51.1)	8 (44.4)	11 (40.7)	0.679
Married/co-habitating	32 (71.1)	6 (33.3)	23 (85.2)	0.001
Completed primary education	31 (68.9)	7 (38.9)	15 (55.6)	0.084
Past/present smoking	14 (31.1)	9 (50)	12 (44.4)	0.297
Number of sexual partners ≥ 4	27 (60)	11 (61.1)	18 (66.7)	0.848
Previous sexually transmitted disease	9 (20)	8 (44.4)	7 (25.9)	0.142
Past use of oral contraceptive	18 (40)	11 (61.1)	18 (66.7)	0.063
Past use of condom	17 (37.8)	3 (16.7)	5 (18.5)	0.105
Pre-conception use of cART	13 (28.9)	2 (11.1)	12 (44.4)	0.056
CD4^+^ T-cell counts > 350 cel/mm^3^ at entry^*a*^	30 (75)	6 (37.5)	11 (40.7)	0.005
CD4^+^ T-cell counts > 350 cel/mm^3^ at delivery^*b*^	33 (84.6)	10 (76.9)	15 (62.5)	0.144
HIV VL (log ≥ 4) at entry^*c*^	12 (28.6)	9 (56.3)	13 (52)	0.065
Codon 72 of *TP53*				0.563
Arg/Arg	18 (40)	5 (27.8)	12 (44.4)	
Arg/Pro	20 (44.4)	7 (38.9)	10 (37)	
Pro/Pro	7 (15.6)	6 (33.3)	5 (18.5)	

*a*: missing CD4 counts at entry, seven patients;*b*: missing CD4 counts at delivery, 14 patients;*c*: missing human immunodeficiency vírus (HIV) viral load (VL) at entry, seven patients; Arg: arginine; cART: combination antiretroviral therapy; in boldface: ignificant p-values at the 0.05 level.

**TABLE IV:** Logistic regression analysis of factors associated with human papillomavirus (HPV) type-specific persistence or re-infection

Variable	Type-specific persistence	p	Re-infection	p
	
	OR		OR	
Age (years)				
< 25	1	0.16	1	0.87
≥ 25	2.08 (0.73-5.90)		0.91 (0.30-2.75)	
Age of first sexual intercourse (years)				
≤ 15	1	**0.03**	1	0.17
> 15	2.96 (1.09-8.08)		2.20 (0.70-6.89)	
Parity				
0- 1	1.52 (0.58-3.99)	0.39	1.30 (0.44-3.92)	0.63
> 1	1		1	
Status marital				
Single/widow/divorced	0.43 (0.12-1.48)	0.17	4.92 (1.52-15.91)	**0.006**
Married/co-habitating	1		1	
Primary education				
Uncompleted	1.77 (0.66-4.75)	0.25	3.48 (1.12-10.86)	**0.03**
Completed	1		1	
Past/present smoking				
No	1	0.25	1	0.16
Yes	1.77 (0.66-4.75)		2.21 (0.72-6.78)	
Number of sexual partners				
1-3	1	0.57	1	0.94
≥ 4	1.33 (0.49-3.62)		1.05 (0.34-3.21)	
Previous sexually transmitted disease				
No	1	0.56	1	0.06
Yes	1.40 (0.45-4.33)		3.20 (0.98-10.44)	
Past use of oral contraceptive				
No	1	**0.03**	1	0.13
Yes	3.0 (1.11-8.14)		2.36 (0.77-7.22)	
Past use of condom				
No	2.67 (0.82-8.38)	0.09	3.03 (0.77-12.05)	0.10
Yes	1		1	
Preconception use of cart				
No	0.51 (0.19-1.38)	0.18	3.25 (0.65-16.18)	0.20
Yes	1		1	
CD4 T-cell at entry^*a*^				
≤ 350	4.36 (1.53-12.46)	**0.005**	5.00 (1.45-17.27)	0.008
> 350	1		1	
CD4 T-cell at delivery^*b*^		0.05		
≤ 350	3.30 ( 0.99-10.95)		1.65 (0.35-7.82)	0.67
> 350	1		1	
HIV VL at entry^*c*^				
Log 1-3	1	0.06	1	**0.05**
Log ≥ 4	2.70 (0.97-7.60)		3.21 (0.97-10.60)	
Codon 72 of p53		0.83		0.27
Arg/Arg	1.33 (0.47-3.82)		0.79 (0.21-2.95)	
Arg/pro	1		1	
Pro/pro	1.43 (0.36-5.66)		2.45 (0.61-9.82)	

*a*: missing CD4 counts at entry, 7 patients;*b*: missing CD4 counts at delivery, 14 patients;*c*: missing human immunodeficiency virus (HIV) viral load (VL) at entry, 7 patients; Arg: arginine; in boldface, significant p-values at the 0.05 level; OR: odds ratio.

*Cytological outcomes based on HPV persistence* - The proportion of patients who showed unfavourable outcomes increased from the nonpersistent (cleared) HPV infections to re-infections and then to type-specific persistence, being the highest in the latter group. In the nonpersistent group, there were six LSIL and two HSIL in the first sample and all cases improved in the second sample. Four new cases of LSIL were seen. In the re-infection group, there were eight LSIL at baseline with two improvements and two new cases of LSIL in the follow-up. In type-specific group, initial sample showed 13 LSIL and two HSIL. In the follow-up, three LSIL evolved to HSIL and four new cases of LSIL developed. The OR was almost three times higher in the type-specific group when compared with the re-infection group (OR = 2.8; 95% CI: 0.43-22.79).

## DISCUSSION

In this study, we analysed 90 HIV/HPV co-infected, pregnant women for rate of persistence of HPV after delivery and risk factors associated with persistence over 18 months. Overall, over 80% of the studied women were infected by HR HPV types at baseline. HPV DNA persistence occurred in 50% of the women after 18 months, but only in 30% showed type-specific persistence. The remaining 18 patients (20%) were rather re-infected by different HPV types, and do not correspond to bonafide HPV persistence. The use of sequencing methodology to classify the different types of HPV has enable us to determine the true rate of infecting HPV types and avoid overestimation of HPV persistence.

Our HPV typing approach has enabled us to evaluate all cases of persistence by individual HPV types. Of note, the relative efficiency of HPV persistence by types 16 and 58 was possible to infer in view of the higher number of cases in our cohort. HPV 58 appears to persist with higher efficiency than HPV 16 (36% vs. 26% of the infections, respectively), although statistically significance has not been achieved. Finally, despite only four cases of infection were observed, two of HPV 18 and two of HPV 56, both types persisted in the period analysed, highlighting its potential in the faster development of cervical abnormalities, as suggested by others (Rintala et al. 2012), and CC. We observed diverse rates of persistence among the 13 LR HPV types, albeit lower than the observed rates for HR types. In two patients, the persistence of LR types occurred with concomitant infection with HR types. Of five patients with exclusively LR HPV type infections (HPV 81 and 62), two had an LSIL lesion in the first cytological exam that persisted and another patient developed LSIL during follow-up (HPV 70). We are currently cloning those cases to exclude the presence of HR types as minority species, and also confirming the cytological exam diagnosis through biopsy, but those results may indicate LR HPV types with enhanced persistence potential.

We find a higher prevalence of *TP53* genotypes encoding proline at codon 72 in our patients (60%) which is in accordance with the genotype among highly mixed populations (Klug et al. 2009). Although no correlation of genotypes with HPV persistence was observed, the small sample size might account for the absence of correlation. In a study with Latin women from Costa Rica, genotypes containing proline (Pro/Arg or Pro/Pro) had an 1.3 and 1.8-fold increased risk for developing grade 3 cervical intraepithelial dysplasia/persistence, respectively, when compared with the Arg/Arg genotype (Koshiol et al. 2009).

An important issue to be considered in HPV persistence studies is the timespan and the definition of persistence. HPV persistence is regularly defined as having two or more HPV DNA positive tests and test intervals greatly vary between studies, complicating comparative analyses (Rositch et al. 2013). Additional factors, such as considering only HR HPV types, and defining type-specific or only HPV DNA for persistence analyses, further preclude accurate HPV persistence estimates. In a recent meta-analysis that included 86 distinct HPV persistence studies, the mean HPV testing interval was 9.8 months, whereas the authors estimate that approximately half of the HPV infections persist past six-12 months. Therefore, we think the test interval used herein (18 months) is appropriate and decreases the chance of overestimating persistence or re-infection.

As mentioned above, HPV persistence has been associated with development of cytological abnormalities and progression to neoplasia. To evaluate the impact of HPV persistence on the progression of cytological abnormalities, we have compared the cytological outcomes of the women throughout the 18-month analysed period. Our study suggests that type-specific HPV persistence has a better predictive value to cervical lesion progression and its use should be reinforced over traditional HPV persistence assays.

The present study has some important limitations. One of them is the lack of histopathologic confirmation of the cytologic abnormalities through biopsy. Another limitation was the low HPV persistence rates which precludes us from performing a detailed analyses of the type-specific and re-infection groups. Nevertheless, we showed the importance of identification of specific HPV types for the discrimination between bonafide persistence and clearance followed by re-infection. Measurements of true incidence of new HPV infections in subjects is a difficult task, because they can be infected by new viruses and clear them over the time period spanning two collection times, and those transitory infections will not be detected. However, by addressing type-specific HPV persistence, we can at least get better estimates of new infections, excluding those cases where the HPV types differ between the two timepoints analysed. Additional re-infection cases may also be excluded if the two viruses are genetically different, even if belonging to the same HPV type, an analysis that requires full-length genome sequencing and phylogenetic estimates, procedures that we did not conduct in this study.

HPV persistence is a risk factor for the development of cervical intraepithelial neoplasia and cervical carcinoma, whereas persistence by HR types is a prerequisite for those conditions (zur Hausen 2000). HIV-infected women are at particularly higher risk of HPV infection; likewise, pregnancy is a condition for increased prevalence of HPV (Hernández-Girón et al. 2005) and for faster progression from SIL to carcinoma (Palle et al. 2000). The contribution of each of these factors to cervical carcinogenesis is not completely understood, but both promote immune suppression and changes in the hormonal milieu (Hernández-Girón et al. 2005), influencing viral acquisition and persistence.

We have found HPV persistence of 50% after delivery in our cohort. Reports of persistence after pregnancy are scarce and it decreases with longer time of follow-up. Castellsagué et al. (2009) found a HPV persistence of 46.2% among pregnant women in six week postpartum samples. Jalil et al. (2013) reported persistence in a later time point (average of 6 months after delivery) of 51.9% and 53.6% among HIV-infected and HIV-uninfected pregnant women for some types. In both reports approximately 28.5% were infected by untyped HPV. Nobbenhuis et al. (2002) reported persistence of 31% for HR types, after an average of six months after delivery. Our study is the first to assess clearance of HPV after delivery describing all types involved in the infection and with a clear definition of bonafide or re-infection persistence.

The HPV type 16 was the most prevalent type in this cohort and type 58 was the second most common type found. In a meta-analysis of HPV prevalence in five continents, type 58 was the third most common type in Latin America and Caribbean. In relation of type-specific persistence, our results confirm previous evidence found in a recent longitudinal survey of the Finish Family HPV Study, where HPV 58 showed a higher persistence time compared to HPV 16 (Louvanto et al. 2010). These results call our attention that type 58 should be included among specific primers used to detect HPV, at least in our region.

Our results corroborate previous report showing that low CD4^+^ T-cell counts are associated with HPV persistence (Kang & Cu-Uvin 2012), and illustrate the important role of cellular immune responses in the control and clearance of HPV infection (Frazer 2009). HIV-infected women with a CD4 < 200 cells/mm^3^ have almost two times more chance of HPV persistence when compared with those with a CD4 T-cell counts > 500 cells/mm^3^ (Ahdieh et al. 2001).

In conclusion, the results herein highlight the importance of HPV persistence in the setting of immunosuppressed patients and their associated risk factors. In addition, efforts to implement true type-specific HPV identification in persistence should be made to avoid overestimation of persistence and to more accurately predict risk for cytological abnormalities and cervical carcinoma in HPV-infected women.
